# Immune Alterations and Viral Reservoir Atlas in SIV-Infected Chinese Rhesus Macaques

**DOI:** 10.3390/idr17010012

**Published:** 2025-02-06

**Authors:** Julien A. Clain, Morgane Picard, Henintsoa Rabezanahary, Sonia André, Steven Boutrais, Ella Goma Matsetse, Juliette Dewatines, Quentin Dueymes, Elise Thiboutot, Gina Racine, Calaiselvy Soundaramourty, Fabrizio Mammano, Pierre Corbeau, Ouafa Zghidi-Abouzid, Jérôme Estaquier

**Affiliations:** 1 Centre Hospitalier Universitaire (CHU) de Québec Centre de Recherche, Faculté de Médecine, Université Laval, Québec, QC G1V 0A6, Canada; julien.clain.1@ulaval.ca (J.A.C.); henintsoa.rabezanaha@crchudequebec.ulaval.ca (H.R.); steven.boutrais@crchudequebec.ulaval.ca (S.B.); aude-ella.goma-matsetse@crchudequebec.ulaval.ca (E.G.M.); juliette.dewat@hotmail.fr (J.D.); quentin.dueymes@crchudequebec.ulaval.ca (Q.D.); elise.thiboutot@crchudequebec.ulaval.ca (E.T.); gina.racine@crchudequebec.ulaval.ca (G.R.); ouafa.zghidi-abouzid@crchudequebec.ulaval.ca (O.Z.-A.); 2 Institut national de la santé et de la recherche médicale (INSERM) U1124, Université Paris Cité, 75006 Paris, France; picard.morgane.noelie@gmail.com (M.P.); smc.andre88@gmail.com (S.A.); selvys93@yahoo.fr (C.S.); fabrizio.mammano@inserm.fr (F.M.); 3 Institut national de la santé et de la recherche médicale (Inserm) U1259 MAVIVHe, Université de Tours, 37032 Tours, France; 4 Institut de Génétique Humaine, CNRS-Université de Montpellier UMR9002, 34094 Montpellier, France; pierre.corbeau@igh.cnrs.fr

**Keywords:** aids, SIV, cure, reservoir, CD4, Tfh, monocyte, B cell, apoptosis, macaque

## Abstract

Background/Objectives: Over the last decades, our projects have been dedicated to clarifying immunopathological and virological events associated with Human Immunodeficiency Virus (HIV) infection. Methods: By using non-human primate models of pathogenic and non-pathogenic lentiviral infections, we aimed at identifying the cells and tissues in which the virus persists, despite antiretroviral therapy (ART). Indeed, the eradication of viral reservoirs is a major challenge for HIV cure. Results: We present a series of results performed in rhesus macaques of Chinese origin deciphering the virological and immunological events associated with ART that can be of interest for people living with HIV. Conclusions: This model could be of interest for understanding in whole body the clinical alteration that persist despite ART.

## 1. Non-Human Primates and SIV Infections

**Figure 1:**
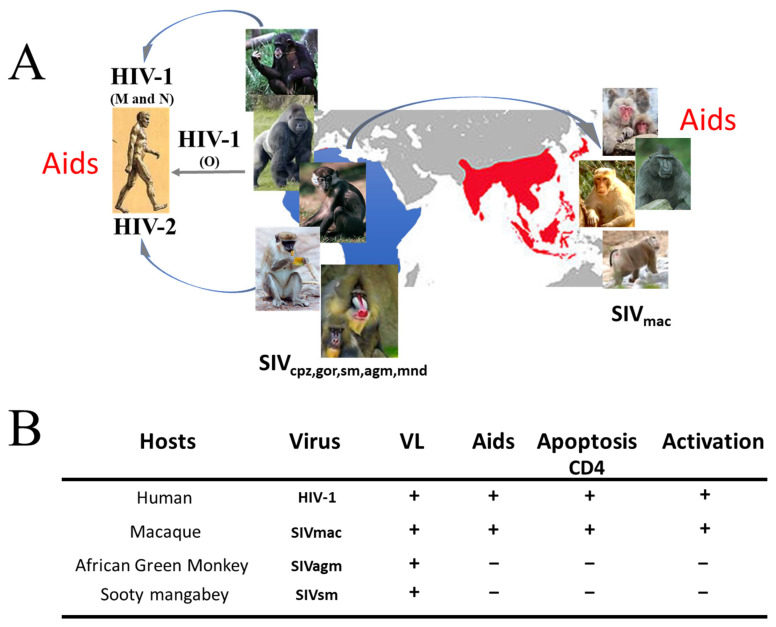
(**A**) The Human Immunodeficiency Virus (HIV-1) pandemic in humans resulted from cross-species transmissions of a strain of Simian Immunodeficiency Virus that infects central African chimpanzees (*Pan troglodytes troglodytes*) (SIV_CPZ_). Thus, SIVcpz is responsible for the emergence of the HIV-1 M and N groups. Furthermore, the infection of gorillas leading to the emergence of SIVgor is responsible for the emergence of the HIV-1 O group (see review [29]). SIVsmm from sooty mangabeys is associated with the emergence of HIV-2 in humans, and cross-species transmission to captive rhesus macaques in US primate centers is responsible for the emergence of the SIVmac. SIVs can also be isolated from mandrills (SIVmnd) and from African green monkeys (SIVagm). Whereas the majority of African NHPs manifest a benign course of natural SIV infection when infected with their species-specific SIV strain, monkeys of Asian origin, such as rhesus *(Macaca mulatta)*, pigtailed *(Macaca nemestrina)*, and cynomolgus *(Macaca fascicularis)* macaques, are susceptible to SIVmac infection. (**B**) Whereas in the blood, viremia is detected in both pathogenic and non-pathogenic non-human primate models of lentiviral infections, only humans and macaques demonstrate T cell immunodeficiency and AIDS. CD4 T cell apoptosis and immune activation characterize pathogenic lentiviral infections.

Simian Immunodeficiency Viruses (SIVs) have been isolated from naturally infected Old World primates, including chimpanzees (SIVcpz) [1], African green monkeys (SIVagm) [2,3], sooty mangabeys (SIVsm) [4], mandrills (SIVmnd) [5,6], and gorillas (SIVgor) [7] (Figure 1). There is generally no acquired immunodeficiency syndrome (AIDS) associated with these strains of SIV in their natural hosts (Figure 1A). However, macaques developing a disease closer to that observed in humans, including the death and depletion of CD4 T cells [8], were transmitted by captive sooty mangabeys housed in United States primates, leading to the isolation of the SIVmac [9] (Figure 1B). Thus, non-human primates (NHP) have been used to decipher the pathogenic events associated with AIDS and to test vaccines and therapies. Rhesus macaques (RMs) (*Macaca mulatta*) of Indian origin are generally used for research purposes. They display higher levels of viral replication, full depletion of CD4 T cells expressing the main co-receptor CCR5, and higher T cell death, compared to cynomolgus macaques (CMs) (*Macaca fascicularis*), which are less sensitive to SIV infection [10] (Table 1). Furthermore, due to the drastic CD4 T cell depletion, almost a third of SIV-infected Indian RMs fail to produce SIV-specific antibodies and to demonstrate an immune activation. Several groups, including ours, have been working with RMs of Chinese origin, which offer several advantages [11,12,13,14,15,16]. In fact, the dynamics of immune events in Chinese RMs are closer to the clinical observations in people living with HIV (PLWH). Indeed, in the blood of PLWH, the proportion of CD4 T cells expressing CCR5 increases following HIV infection [17,18,19,20]. This increase is a marker of disease progression, particularly because immune activation promotes the expression of CCR5 [17,18,19,20]. Accordingly, the absence of CCR5 increases in African green monkeys during the acute phase is associated with a low level of T cell immune activation, contrary to what is observed in SIV-infected RMs [16]. Whereas a drastic depletion of CCR5-positive T cells is reported in Indian RMs [21,22], this depletion in the blood of RMs of Chinese origin is transient, and this proportion is increasing post-acute phase [13], consistent with the dynamics observed in PLWH [17,18,19,20]. We have also reported that in naïve RMs of Chinese origin [13], the percentages of CD4 T cells expressing CCR5 (approximately 7%) are similar to those observed in non-pathogenic NHP models like in African green monkeys or sooty mangabeys and different from that reported for CD4 T cells of Indian RMs (approximately 20%) [21,22,23] (Table 1). Thus, this observation in RMs of Chinese origin suggests that the loss of memory CD4 T cells cannot be solely attributed to a difference in CCR5 expression between pathogenic and non-pathogenic NHP models. Although CCR5 represents one of the main co-receptors, we have previously found that alternative co-receptors such as Bob/GPR15 may participate in the priming of T cells from RMs to die by apoptosis; however, the dynamics of CD4 T cells expressing this co-receptor remain poorly studied [24]. Another animal model is the pigtailed macaque (*Macaca nemestrina*), which is highly susceptible to SIV infection [25], showing a drastic CD4 T cell depletion, the absence of SIV-specific antibodies [26], and developing encephalitis within a couple of weeks after infection [27]. Therefore, this animal model is less useful in designing effective antibody-based vaccines or in evaluating the correlates of protection associated with humoral immunity. However, whereas this phenotype is generally observed in US colonies, pigtailed monkeys living in Australia display a distinct profile in which a specific humoral response is observed [28]. This difference may reflect genetic inbreeding in the US, related to the limited number of founders.

Indeed, a number of studies have revealed that certain macaque major histocompatibility complex (MHC; Mamu) class I alleles, including Mamu-A*01 [30], Mamu-B*01 [31], and Mamu-B*17 [32], among others [33] are expressed with high frequency in Indian RM populations. The beneficial impact of TRIM5 alleles in Mauritian CMs also demonstrates limited genetic diversity due to a small number of founder animals with only seven MHC haplotypes (M1-M7) [34]; such diversity is higher in other breeding colonies [35] (Table 1). These MHC class I alleles are more polymorphic in Chinese RMs than in their Indian counterparts, and few are overlapping [36,37,38,39]. The Mamu-A1*02201, one of the most frequent alleles identified, is an analog to HLA-B7 supertype in humans [37]. The Mamu-A1* 02601 and Mamu-B*08301 alleles, each representing a frequency of 6%, share characteristics in terms of peptide antigen recognition with the HLA-A2 and HLA-A3 supertypes in humans. Thus, these three common Mamu class I alleles identified in Chinese RMs, which are absent in Indian RMs [37,40,41], are associated with a peptide motif corresponding to one of the three most common HLA supertypes expressed in humans. When HLA-B7, HLA-A2, and HLA-A3 supertypes are combined, over 86% of the human population is covered [42]. These differences between monkey species could likely be the consequence of the USA moratorium on animal import in 1978 and the subsequent inbreeding in primate centers.

Furthermore, in Indian RMs, in addition to the restricting Tripartite motif 5α factor (TRIM5α) in which TFP/TFP and TFP/Q genotypes are predominant [43], the Mamu-B*08 [44,45], Mamu-B*17 and Mamu-A*01 alleles are associated with viral control [32,46,47] (Table 1). Peptides presented by Mamu-B*08 share a binding motif with peptides presented by HLA-B*27 allele, which is associated with viral control in humans [44,48,49,50]. In the German Primate Center breeding colony, the average frequency of the Mamu-A*08 allele was reported to be 48% [51]. Whereas the HLA-B*57 allele has been identified earlier in HIV-1-infected long-term non-progressors [52,53,54,55], little is known so far regarding its analog in macaques. In CMs, the M1 and M6 MHC haplotypes have also been mostly associated with spontaneous SIV control [56,57,58]. However, the beneficial impact of TRIM5α alleles on SIV-infection remains controversial [59,60].

Therefore, based on our expertise with SIVmac-infected Chinese RMs, we have developed this model to assess a viral reservoir (VR) atlas after antiretroviral therapy (ART) administration.

## 2. Viral Dynamics and Persistence Despite ART

Viral production in HIV-infected individuals results from a dynamic process involving continuous rounds of de novo infection and replication in CD4 T cells, along with the rapid turnover of both cell-free virus and virus-producing cells. The level of viral load is a strong predictor of disease progression [61,62,63]. However, assessing antiretroviral therapies in monkey models also needs to consider the strains of SIV used and the age of the animals. Indeed, different SIV strains are used to infect RMs, such as SIVmac239 and SIVmac251; although the former is less potent in infecting the macrophages in vitro [64]. NHP infection may also be carried out with simian/human immunodeficiency viruses (SHIVs) that express the HIV envelope but often yield too low viral loads and fail to establish persistent viral infection after 3 months [65]. This attenuated profile is exacerbated in CMs. Similarly, a nef-deleted SIVmac is associated with low viral loads and is generally considered non-pathogenic in macaques [66]. However, this attenuated virus can lead to AIDS in a large proportion of neonatal and adult RMs after several years [67,68,69].

**Figure 2:**
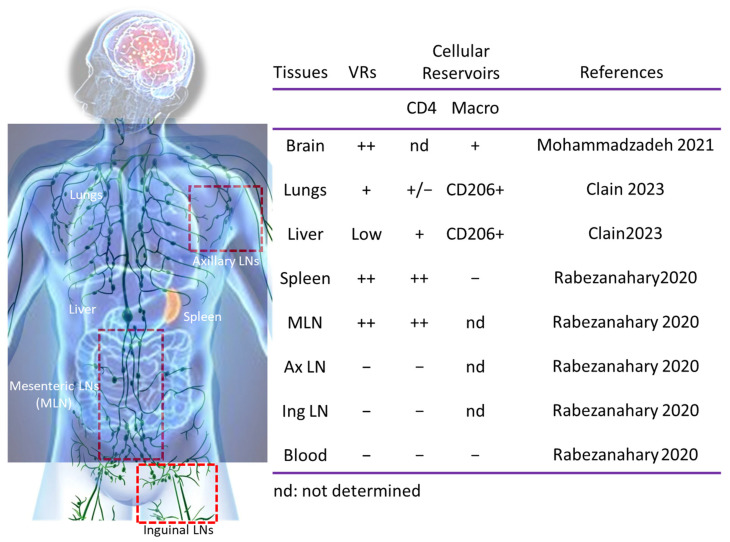
Impact of early antiretroviral therapy on the establishment of tissue and cellular reservoirs in SIVmac251-infected rhesus macaques of Chinese origin. Tissue and cellular atlas of viral reservoirs (VRs) in SIVmac251-infected RMs of Chinese origin. From the same individuals, an extensive exploration of lymphoid and myeloid VRs has been performed in blood [80,81] and from different tissues such as brain [82], lung [83], liver [83,84], spleen [80,81], axillary and inguinal lymph nodes (Ax LN and Ing LN) [80] and mesenteric lymph nodes (MLN) [80]. The presence (+) and the absence (−) of VRs is indicated. The nature of cells in which viral DNA has been detected is indicated: macrophages (Macro) and CD4 T cells (CD4). Nd: Not determined at the time of this publication. The references of the different manuscripts in which the results have been published are indicated.

With the advent of antiretroviral combined therapy, the eradication of VRs remains a major challenge for HIV cure [70]. In most PLWH, plasma viral rebound occurs within days or weeks after ART interruption (ATi) [71,72,73,74,75]. However, due to the challenges of sampling tissues in humans, macaques have been useful in deciphering the early events associated with viral dissemination and addressing the establishment of VRs under ART. ART administered early after infection before plasma viremia detection [76] is unable to prevent VR establishment [77,78,79]. This initial observation by Whitney et al. [76], reporting the absence of viral detection in blood and peripheral LNs in Indian RMs, was of importance, indicating that the source of viral rebound after ATi was elsewhere [76,80]. Therefore, we performed similar experiments in Chinese RMs infected with SIVmac251 and treated with early ART (Figure 2). We reported that VRs are seeded in mesenteric lymph nodes (MLNs) and in the spleen [80].

The establishment of VRs also generates a large fraction of cells with defective genomes, which represent the vast majority of HIV-1 DNA persisting under ART [85,86,87]. Although defective, these genomes may lead to the expression of some viral antigens, triggering cytolytic immune responses [88,89]. This viral persistence may also contribute to HIV-associated inflammation through the recognition of HIV proteins and RNAs that activate innate antiviral responses [90,91]. Recent studies in SIVmac239- and SHIV-infected Indian RMs treated with ART have shown the persistence of defective proviruses as well [92]. Of importance was the observation obtained using immunoPET (antibody-targeted positron emission tomography) showing that in aviremic antiretroviral-treated Indian RMs, the glycoprotein of SIV is detectable in various tissues, including the colon, certain lymph nodes, and lungs [93], indicating the presence of transcriptionally active infected cells despite ART.

The quality of immune cells in maintaining viral control within tissues under ART is crucial for effective viral suppression [94,95,96]. Recently, it has been shown that combining ART with a therapeutic strategy based on anti-IL-10 and anti-PD-1 monoclonal antibodies improves T cell immune response in SIV-infected Indian RMs and reduces viral rebound after ATi [97]. This observation is consistent with the suppressive role of IL-10, which is of critical importance during the acute phase [98,99]. By counter-balancing the positive action of IL-12, as described more than two decades ago [98,99,100], IL-10 could contribute to NK cell impairment and hinder viral clearance. Indeed, NK cells have been proposed to play an important role in eliminating productive infected cells in the lymph nodes of SIV-infected African green monkeys [101].

Thus, immunotherapies in association with ART offer promising new avenues to boost the immune system’s ability to tackle anatomical VRs.

## 3. Myeloid Cells and VRs

Blood monocytes consist of subsets with distinct phenotypic and functional characteristics. The expression of CD14 (lipopolysaccharide [LPS] coreceptor) and CD16 (FcγRIII) distinguishes classical (CD14++ CD16−), intermediate (CD14++ CD16+), and nonclassical (CD14+/− CD16+) monocyte subsets [102]. Whereas the CD14 population expresses the chemokine receptor CCR1 and CCR2, enabling migration into inflamed tissues via CCL2 and CCL3 gradients, the CD16 subset expresses CX3CR1 and migrates into the inflamed tissues in response to CX3CL1. Blood monocytes also express CD4 and CCR5, which are important for HIV infection [103,104]. In this context, although blood monocytes are non-cycling and non-proliferating cells, productive infection coincides with their entry into the G1/S phase of the cell cycle. Granulocyte-macrophage colony-stimulating factor (GM-CSF) is one of the main cytokines that promotes and sustains their productive infection [105,106,107]. Myeloid cells support high levels of viral replication, especially during bacterial infection [108] or when T cells have been depleted [109,110].

Whereas viral DNA can be detected in blood monocytes [111,112,113], we and several groups have reported the beneficial effect of ART in reducing the pool of infected monocytes [114,115,116,117]. In a model of humanized mice, persistent infection of myeloid cells is responsible for viral rebound after ATi [118]. The pool of infected blood monocytes is drastically reduced when ART is administrated early after infection in SIVmac251-infected RMs of Chinese origin [81] (Figure 2). This is critical, as monocytes contribute to the turnover of intestinal macrophages during inflammation and after tissue injury [119,120]. Furthermore, we have reported that early ART prevents the infection of monocytes in the spleen. This is of importance, given that splenic monocytes represent major cellular reservoirs mobilized early after trauma [121]. Therefore, by preventing monocyte infection, ART has a beneficial effect in limiting viral dissemination.

Inflamed and resident macrophages [122] may also contribute to the establishment of VRs. Thus, viral DNA also persists in pulmonary macrophages of ART-treated pigtailed macaques [123] and in PLWH associated with immune pulmonary alterations [124,125,126]. Recently, our team found that pulmonary CD206+ tissue-resident macrophages contain viral DNA despite early ART in SIVmac251-infected RMs of Chinese origin [83]. Infection of macrophages has also been earlier established in the brain [127], including perivascular cells [128] associated with cognitive impairment in SIV-infected RMs [129]. Viral DNA and neurocognitive symptoms have also been described in PLWH despite ART [130,131]. Thus, the brain from different macaque species was shown to harbor SIV-infected cells [82,132,133] even when treated with early ART. Importantly, our study in early ART-treated SIVmac251-infected Chinese RMs revealed that brain tissues harbor significant levels of viral RNA/DNA and capsid antigens in microglia/macrophages [82]. The liver may also contain a persisting virus under ART [134], in which, similarly to the lungs, the CD206+ tissue-resident macrophages harbor viral DNA [83] (Figure 2).

In both the brain and lungs, an inflammatory signature persists, despite early ART administered in SIVmac251-infected Chinese RMs [83,135]. This is particularly significant given that chronic inflammation is associated with comorbidities in PLWH [136,137,138,139]. In this context of inflammation, it has been shown that ART is unable to reduce the expression of caspase-1, the main effector molecule of the inflammasome, and IL-18 [140,141]. IL-18, which is an inducer of FasL, a pro-apoptotic molecule [142,143], is a substrate of caspase-1 [144,145]. The administration of Q-VD, a broad-spectrum caspase inhibitor, effectively inhibits caspase activation and reduces inflammation in SIVmac251-infected RMs of Chinese origin [146]. Other inhibitors could be of interest, such as z-VAD-FMK, but this molecule interferes with cell proliferation and maturation [147,148], or VX-765, which is specific to caspase-1 [149,150]. Given that chronic inflammation is a characteristic of PLWH, these molecules might offer potential benefits for PLWH undergoing ART.

## 4. CD4 T Cell Subsets and VRs

**Figure 3:**
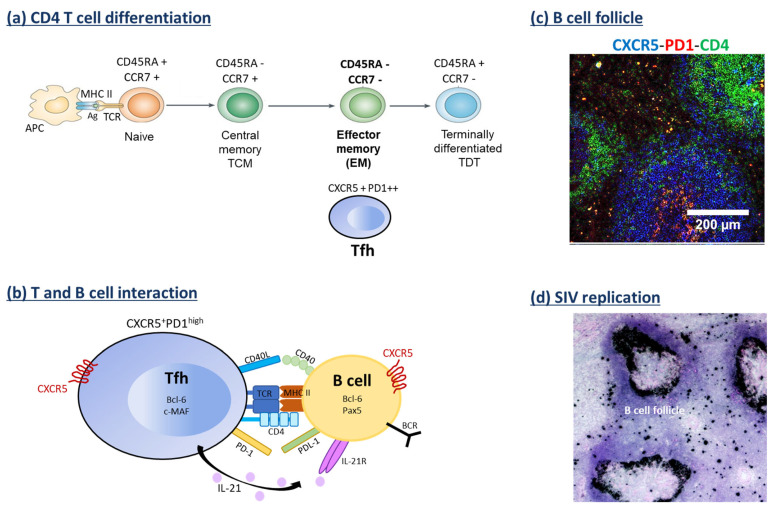
T follicular helper cells and Simian Immunodeficiency Virus infection. (**a**) CD4 T cell differentiation. After antigen (Ag) presentation by the major histocompatibility complex (MHC) class II molecules expressed by antigen-presenting cells (APC) to the T cells expressing the T cell receptor (TCR), naïve T cells are activated, leading to their differentiation into central memory (CM), effector memory (EM) or terminally differentiated T (TDT) cells, as defined by the expression of CD45RA and CCR7 molecules. In addition, among EM, there is a subset of CD4 T cells, namely T follicular helper (Tfh) cells, that express the C-X-C chemokine receptor type 5 (CXCR5) and the programmed cell death protein 1 (PD-1) molecules. (**b**) T and B cell interaction. Tfh cells are essential for B cells by providing co-signals (CD40 and PDL-1) leading to the expression of transcriptional factors such as Bcl-6 (B-cell lymphoma 6) and Pax5 (Paired box protein 5) in B cells. In turn, B cell interaction provided co-signal to Tfh cells in inducing the transcription factors Bcl-6 and c-MAF, (musculoaponeurotic fibrosarcoma), which in turn led to the production of the interleukin 21 (IL-21) and sustained B cell activation and maturation. (**c**) B cell follicle. The formation of germinal centers depends at least in part on the interaction of Tfh and B cells. Confocal microscopy shows CXCR5, PD-1, and CD4 expressions from the spleen of a naïve rhesus macaque. (**d**) SIV replication. Viral replication is determined by in situ hybridization using a specific SIV nef probe (dark spots) demonstrating strong staining in the region of B cell follicles, as well as the accumulation of viral RNA in the follicular dendritic cell (FDC) network that may represent viral particles trapped at the surface of the FDCs.

CD4 T cells are crucial to achieving a regulated, effective immune response to pathogens. They may acquire distinct cytokine polarization (e.g., Th1, Th17, Treg) controlled by inflammation and metabolism [151]. Thus, naïve CD4 T cells are activated upon interaction with the antigen-MHC complex and differentiate into specific subtypes, mainly depending on the cytokine milieu of the microenvironment (Figure 3). Central memory (TCM) and transitional memory (TTM) CD4 T lymphocytes have been reported to be the main VRs in the blood of PLWH [152]. While a role of long-lived stem cell memory CD4 T cells has been proposed [153], other groups have reported a significant enrichment in TCM expressing the chemokine receptor CCR6 in PLWH as VRs [154], considered to be related to Th17 [151]. Whereas this population has been described in SIV-infected RMs of Indian origin [155], another subset of memory CD4 T cells expressing CTLA-4 but lacking programmed death-1 (PD-1) expression has also been reported as VR, sharing markers with regulatory T cells [156]. This may reflect the altered balance of Th17/Treg cells since Th17 cells are selectively depleted from the gut [157,158]. However, we have observed that such balance is partially restored after an early ART in SIVmac251-infected RMs of Chinese origin in lymph nodes draining the large and small intestines [159]. These exhausted molecules, CTLA-4 and PD-1, have been proposed to promote HIV latency in PLWH [160,161]. In non-lymphoid tissues such as the liver, memory CD4 T cells, which strongly express CCR5, are also infected and depleted in SIVmac251-infected RMs of Chinese origin. These cells undergo transcriptional reprogramming, with increased expression of granzyme A and members of the transforming growth factor (TGF)-β family, potentially contributing to fueling the hepatic inflammation and fibrosis [84]. However, the role of CD4 T cells as VRs in the hepatic tissues of PLWH remains poorly addressed so far.

In addition, the T follicular helper (Tfh) cell subset, which produces IL-21 and is essential for germinal center (GC) development, B cell maturation, and antibody production [162,163,164,165], has also been identified as a VR [28,166,167,168,169,170,171] (Figure 2). This population highly expresses the chemokine receptor CXCR5 and PD-1 (Figure 3) [164,165]. Although an expansion of circulating Tfh has been reported [172,173], the percentages of Tfh cells in peripheral LNs from progressor RMs are lower than in non-progressor RMs [168,174]. Furthermore, we have reported an early depletion of Tfh cells after infection, both in the spleen and MLNs of SIVmac251-infected RMs of Chinese origin [171,175]. In addition, this depletion was associated with altered CXCR5 ligand (CXCL13) expression in B cell follicles and profound remodeling of the GC architecture in this model [171,175].

Several transcriptional factors (TFs), both activators [176,177,178,179,180] and repressors [181,182,183,184], play a role in the regulation of Tfh cells. Higher levels of the TFs Foxo1 and KLF2 [181,182,183,184], as well as Stat5 phosphorylation [185,186,187] are associated with a block in Tfh differentiation. In the context of HIV/SIV infections, Tfh is abnormally differentiated [167,171,175,188,189,190,191]. Tfh cells from both the spleen and MLNs of SIVmac251-infected RMs of Chinese origin display a Th1-like profile [171,175] similar to that reported in peripheral LNs of SIV-infected RMs of Indian origin [192]. Given the immunological role of Tfh cells, along with the localization of SIV RNA in the region of B cell follicles and its accumulation in the follicular dendritic cell network (Figure 3), strategies aimed at improving and/or reprogramming Tfh cell function, as well as preventing their infection, could be highly beneficial for PLWH.

## 5. Viral Dissemination After ART Interruption

Once viremia is controlled by early ART and despite the absence of viral detection in the blood and peripheral LNs, a viral rebound is observed within two weeks after ATi [71,72,73,74,75]. Viral rebound can be used as a measure of in vivo inducible VRs and treatment efficacy. Thus, different strategies aiming to tackle VRs are measuring viral rebound after treatment [97]. To assess viral dissemination, we performed several analyses from SIVmac251-infected RMs of Chinese origin that were sacrificed on days 10, 12, 15, and 18 post-ATi. These time-points correspond to 3 days after the first detectable viremia in the blood. Thus, a short time after viral detection, we performed necropsies. Whereas on day 10, viral DNA was detected only in splenic Tfh, all T cell subsets from MLNs displayed viral DNA; none of the CD4 T cell subsets in peripheral LNs expressed viral DNA, indicating a limited dissemination. Nevertheless, in less than 2 weeks, in 3 RMs of Chinese origin (necropsied on days 12, 15, and 18 post ATi), effector memory CD4 T cells (TEM) and Tfh cells expressed viral DNA, both in the spleen and MLNs, but also in peripheral LNs. In these compartments, CM CD4 T cells are also infected, indicating the rapid dissemination of SIVmac251 [76]. Altogether, these observations suggest that once the ART is interrupted, SIV-infected cells are de novo transcriptionally active in MLN and the spleen is capable of generating, in a week, millions of viral particles in the blood. Understanding the role of these populations is of central interest for the cure strategies.

From the same RMs, we also analyzed monocytes across the blood, spleen, and intestine. Whereas no virus was detected in these compartments among individuals necropsied under ART [57], monocytes were infected, expressing viral RNA following ATi. Notably, the spleen emerged as one of the main anatomical sites of viral rebound in SIVmac251-infected RMs of Chinese origin [81]. Thus, on day 10 post-ATi, we detected a few SIV-DNA-positive CD14 cells and mostly localized in the intestine. However, on days 12, 15, and 18 post-ATi, we observed viral DNA in the three different compartments of the necropsied RMs. The main infected monocytic subpopulation was CD14-positive rather than CD14-negative. Nevertheless, the extent of viral infection in monocytes was lower compared to that in CD4 T cells.

Altogether, these results indicate that after ATi, viral dissemination is rapid, targeting CD4 T cells, in which Tfh cells represent the preferentially infected population, but also myeloid cells in SIVmac251-infected RMs of Chinese origin. Thus, different strategies aiming to tackle VRs need to consider the nature of the infected cells after the viral rebound, particularly in tissues where Tfh cells are predominant targets.

## 6. B Cell Response and VRs

As indicated Tfh cells represent the main VRs in visceral lymphoid tissues. A defect in the interaction between B and Tfh cells may lead to an impaired immune response [162,163,164,165]. It has been shown that persistent triggering of PD-1 affects Tfh ability to provide adequate B cell help [190]. The comparison of pathogenic and non-pathogenic lentiviral infections has indicated a negative correlation between the levels of CD4 T cell death and the capacity to generate an IgG response [16,193]. Furthermore, a loss of B cells and an absence of seroconversion were observed in RMs that progress to AIDS, in particular in Indian RMs progressing faster to AIDS, showing an absence of immune activation and a huge depletion of memory CD4 T cells shortly after infection associated with T cell apoptosis [193,194]. Importantly, in B-cell areas surrounding GCs where Tfh are localized, dying B cells have been reported to be associated with GC involution [195]. We have found an early defection in the differentiation of B cells, not only in the spleen but also in MLNs of SIVmac251-infected RMs of Chinese origin, concomitantly with the depletion of Tfh cells [171,175]. Thus, the genesis of mature B cells and survival associated with the production of specific antibodies against HIV positively correlate with the frequency and quality of Tfh cells [174,196].

B cell-activating factor (BAFF) and a proliferation-inducing ligand (APRIL), which shares two receptors with BAFF, play essential roles in B cell regulation. Interestingly, APRIL has been proposed to correlate with the magnitude of vaccine responses [197,198].

Early ART has been reported to preserve peripheral blood B cells in PLWH [199]. However, BAFF levels remain elevated in ART-treated individuals compared to healthy donors [200] and are associated with HIV-related disease progression [201]. Moreover, abnormal B cell differentiation persists in PLWH, and the ability to respond to vaccines often remains compromised [202,203]. Importantly, it has been shown that follicular hyperplasia is not completely resolved following ART [204,205,206], and the amount of LN fibrosis negatively correlates with the vaccine response [207]. This defect in lymphoid tissues may provide a rationale for the observation that Tfh cell function remains impaired in PLWH who do not respond efficiently to the influenza vaccine, despite ART [208,209].

Identifying strategies that may enhance B cell survival and restore Tfh functions could be of interest to PLWH, particularly to improve vaccine response.

## 7. Apoptosis, Caspases and Therapy

Depletion of CD4 T cells, in particular memory, is characteristic of pathogenic lentiviral infections. Studies conducted in both pathogenic and non-pathogenic models of SIV infection demonstrated a direct correlation between progression to AIDS and levels of CD4 T cell apoptosis [8,16,24,98,100,193,210,211,212,213,214,215,216] (Figure 1B). Although viral replication may induce T cell death [217,218,219], most dying cells are non-infected suggesting alternative mechanisms [211,216]. Two main physiological mechanisms contribute to regulating lymphoid cell death: activation-induced cell death (AICD) and death by neglect (or cytokine deprivation). AICD depends on Fas (CD95) and is associated with the activation of caspases, whereas death by neglect activates members of the Bcl-2 family and the mitochondrial pathway of apoptosis [220,221,222]. CD4 T cell death during HIV/SIV infections can be related, at least in part, to heightened levels of immune activation [210,212,213,223,224] and an increased sensitivity to Fas signaling, leading to caspase activation and death [98,100,214,225]. The HIV-1 envelope glycoprotein also induces death [226,227,228,229,230,231,232,233,234,235] through the activation of caspase-3 and caspase-8 [229,230]. CD4 T cells may also die via a caspase-1–mediated pyroptosis [231,236] or by autophagy [237]. A recent report indicates that CARD8 inflammasome drives CD4 T cell depletion during pathogenic HIV/SIV infections [238]. Finally, it was shown that CD4 T cells from aviremic PLWH remain more susceptible to dying by apoptosis compared to healthy donors [239,240,241].

The fact that T cell death remains elevated in PLWH is not anecdotic. Indeed, TEM cells and the more differentiated T cell subsets (TDT) are the subsets more prone to dying by AICD [98,214] compared to TCM and naïve CD4 T cells. While viral DNA detected in TEM is higher compared to TCM, TEM cells are less potent in reactivating integrated viral DNA compared to TCM. This difference in the susceptibility of T cells to die after stimulation is generally not considered in most of the recent studies assessing VRs. Indeed, it cannot be excluded that the inability to detect viral RNA after reactivation is related to the fact that these T cells are dying before being productive. Similarly, attempts to monitor the antigen-specificity of CD4 T cells could also be impacted by AICD. This process contributed to the defective recall immune responses observed in PLWH during the 1990s [242,243]. Counterintuitively, individuals with reduced VRs and T cells less susceptible to apoptosis would be expected to exhibit stronger specific immune responses compared to those with larger VRs and T cells more prone to cell death. Therefore, the modus operandi needs to consider T cell death, even in ART-treated individuals in whom the persistence of VR may impact T cell immunity.

However, the formal proof of the importance of apoptosis was provided by the administration of Q-VD, a broad caspase inhibitor, during the acute phase in the absence of ART. Indeed, we showed that in SIVmac251-infected Chinese RMs treated with Q-VD, the prevention of memory CD4 T cell death enabled the generation of antigen-specific memory CD4 T cells [146]. Most importantly, viremia and cell-associated SIV DNA were reduced and progression to AIDS was delayed in Q-VD-treated RMs compared to control RMs, although we used a model in which vpx contributes to viral virulence [244,245].

Thus, caspase inhibitors could represent an adjunctive therapeutic agent to reduce the VRs and inflammation in PLWH.

## 8. Conclusions

Altogether, these studies have provided strong information regarding cellular and tissue reservoirs for SIV. This is of importance for PLWH, in which such analyses are extremely complicated to perform. Furthermore, these experiments have provided major advances regarding our knowledge of the alterations of the immune system in the course of lentiviral infections, which persist despite early ART. Indeed, virus-induced reprogramming of immune cells occurs early after infection, and strategies aiming to rejuvenate the immune system are needed.

## Figures and Tables

**Table 1:** Comparative analysis of disease progression and immunological features across non-human primate models and humans. RMs: rhesus macaques; PLWH: people living with HIV; peripheral lymph nodes: PLN; C-C chemokine receptor type 5: CCR5; MHC: major histocompatibility complex. The “+” signs indicate the level of viremia: +++: high, ++: moderate and +: low. The “*”refers to the allele’s specific nomenclature in the context of the major histocompatibility complex (MHC). For example, in Mamu-A*01, “Mamu” refers to the rhesus macaque MHC, “A” is the gene locus, and “*01” specifies the allele variant.

	Features	RMs of Indian Origin	RMs of Chinese Origin	Pigtail Macaques (United States)	Cynomolgus (Mauritian)	African Green Monkeys	PLWH
Virus and Disease progression	Blood Viremia PLN Viremia	+++ +++	++ ++	+++ +++	++ +	++ No	++ ++
	Progression to AIDS	Rapid Less than 1 year	Moderate 1 to 5 years	Rapid Less than 6 Months	Low Controller	No Controller	Moderate 5 to 10 years
Immunological parameters	CD4 T depletion	Fast	Moderate	Fast	Low	No	Gradual depletion
	Immune activation	Low	Moderate to High	Low	Low	Low	Moderate to high
	CCR5 expression and after infection	High Full depletion	Low Increase	High Full depletion	Low No increase	Low No increase	Low Increase
	SIV-specific antibodies	Low	High	Low	Low	Low	High
	MHC molecules (most frequent)	Mamu-A*01, Mamu-B*01, Mamu-B*17	Mamu-A*02 Mamu-B*08	Mane-A* 10	Mafa, M1 to M7	Chae-A and Chae-B	HLA-A2, HLA-A3 and HLA-B7

## Data Availability

No new data generated or analysed.
